# 2,3-Bis[(2-cyano­eth­yl)sulfan­yl]-1,4,5,8-tetra­thia­fulvalene-6,7-dicarbonitrile

**DOI:** 10.1107/S1600536810028473

**Published:** 2010-07-21

**Authors:** Cui-Ping Jiang, Bao Li, Bing-Zhu Yin, Li-Xin Wu

**Affiliations:** a Key Laboratory of Organism Functional Factors of the Changbai Moutain, Yanbian University, Ministry of Education, Yanji 133002, People’s Republic of China; b State Key Laboratory of Supramolecular Structure and Materials, College of Chemistry, Jilin University, Changchun 130012, People’s Republic of China

## Abstract

In the title compound, C_14_H_8_N_4_S_6_, the two five-membered rings lie in the same plane with an r.m.s. deviation of 0.0334 (5) Å. The crystal structure features inter­molecular S⋯N inter­actions of 3.295 (4) Å.

## Related literature

For background to the electrical properties of tetra­thia­fulvalene derivatives, see: Fabre (2000 ▶); Batail (2004 ▶). For the synthesis, see Chen *et al.* (2005 ▶). For a related structure, see: Hou *et al.* (2010 ▶).
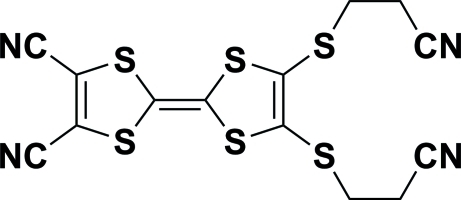


## Experimental

### 

#### Crystal data

C_14_H_8_N_4_S_6_
*M* *_r_* = 424.66
Triclinic, 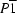

*a* = 7.3055 (15) Å
*b* = 8.5193 (17) Å
*c* = 15.386 (3) Å
α = 82.52 (3)°
β = 76.97 (3)°
γ = 72.43 (3)°
*V* = 887.4 (3) Å^3^
*Z* = 2
Mo *K*α radiation
μ = 0.77 mm^−1^
*T* = 290 K
0.13 × 0.12 × 0.11 mm

#### Data collection

Rigaku R-AXIS RAPID diffractometer
Absorption correction: multi-scan (*ABSCOR*; Higashi, 1995 ▶) *T*_min_ = 0.906, *T*_max_ = 0.920
8751 measured reflections
4020 independent reflections
3415 reflections with *I* > 2σ(*I*)
*R*_int_ = 0.021

#### Refinement

*R*[*F* ^2^ > 2σ(*F* ^2^)] = 0.026
*wR*(*F* ^2^) = 0.072
*S* = 1.09
4020 reflections
217 parameters
H-atom parameters constrained
Δρ_max_ = 0.35 e Å^−3^
Δρ_min_ = −0.20 e Å^−3^

### 

Data collection: *RAPID-AUTO* (Rigaku, 1998 ▶); cell refinement: *RAPID-AUTO*; data reduction: *CrystalStructure* (Rigaku/MSC, 2002 ▶); program(s) used to solve structure: *SHELXS97* (Sheldrick, 2008 ▶); program(s) used to refine structure: *SHELXL97* (Sheldrick, 2008 ▶); molecular graphics: *PLATON* (Spek, 2009 ▶); software used to prepare material for publication: *SHELXL97*.

## Supplementary Material

Crystal structure: contains datablocks global, I. DOI: 10.1107/S1600536810028473/ng2799sup1.cif

Structure factors: contains datablocks I. DOI: 10.1107/S1600536810028473/ng2799Isup2.hkl

Additional supplementary materials:  crystallographic information; 3D view; checkCIF report

## Figures and Tables

**Fig. 1.**
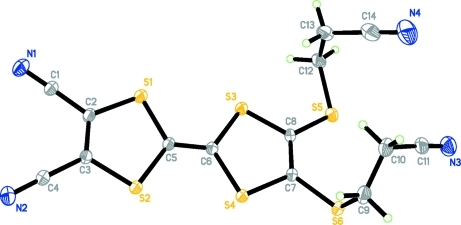
The asymmetric of title compound, with the atom numbering. Displacement ellipsoids of non-H atoms are drawn at the 30% probalility level.

## supplementary crystallographic information

### Comment

The derivants of tetrathiafulvalene (TTF), especially functionalized with hydroxyl or amine groups, have been studies extensively for their interesting electrical properties (Fabre, 2000; Batail, 2004). Herein, we report the crystal structure of the title compound.

The title compound, as shown in Fig. 1, all bond lengths and angles are normal and comparable with those reported for the related structure (Hou *et al.*, 2010). In the crystal, the two five-membered rings lie in the same plane with an r.m.s. deviation of 0.0334 (5) Å. The intermolecular S···N interactions with the distance of 3.295 (4) Å link the molecules into one-dimensional chain along a+c dirction.

### Experimental

The title compound was synthesized as described in the literature for the analogous compound 2,3-Bis(butylthio)-6,7-dicarbonitrile-1,4,5,8- tetrathiafulvalene (Chen *et al.*, 2005). Single crystals and single crystals suitable for X-ray diffraction were prepared by slow evaporation a mixture of dichloromethane and petroleum (60–90 °C) at room temperature.

### Refinement

Carbon-bound H-atoms were placed in calculated positions with C—H = 0.97 Å and were included in the refinement in the riding model with *U*_iso_(H) = 1.2 *U*_eq_(C).

### Crystal data

**Table:**

C_14_H_8_N_4_S_6_	*Z* = 2
*M**_r_* = 424.66	*F*(000) = 432
Triclinic, *P*1	*D*_x_ = 1.589 Mg m^−^^3^
Hall symbol: -P 1	Mo *K*α radiation, λ = 0.71073 Å
*a* = 7.3055 (15) Å	Cell parameters from 7465 reflections
*b* = 8.5193 (17) Å	θ = 3.0–27.5°
*c* = 15.386 (3) Å	µ = 0.77 mm^−^^1^
α = 82.52 (3)°	*T* = 290 K
β = 76.97 (3)°	Block, black
γ = 72.43 (3)°	0.13 × 0.12 × 0.11 mm
*V* = 887.4 (3) Å^3^	

### Data collection

**Table:**

Rigaku R-AXIS RAPID diffractometer	4020 independent reflections
Radiation source: fine-focus sealed tube	3415 reflections with *I* > 2σ(*I*)
graphite	*R*_int_ = 0.021
ω scans	θ_max_ = 27.5°, θ_min_ = 3.0°
Absorption correction: multi-scan (*ABSCOR*; Higashi, 1995)	*h* = −9→9
*T*_min_ = 0.906, *T*_max_ = 0.920	*k* = −11→9
8751 measured reflections	*l* = −19→19

### Refinement

**Table:**

Refinement on *F*^2^	Primary atom site location: structure-invariant direct methods
Least-squares matrix: full	Secondary atom site location: difference Fourier map
*R*[*F*^2^ > 2σ(*F*^2^)] = 0.026	Hydrogen site location: inferred from neighbouring sites
*wR*(*F*^2^) = 0.072	H-atom parameters constrained
*S* = 1.09	*w* = 1/[σ^2^(*F*_o_^2^) + (0.0391*P*)^2^ + 0.0566*P*] where *P* = (*F*_o_^2^ + 2*F*_c_^2^)/3
4020 reflections	(Δ/σ)_max_ = 0.001
217 parameters	Δρ_max_ = 0.35 e Å^−^^3^
0 restraints	Δρ_min_ = −0.20 e Å^−^^3^

### Special details

**Table:**

Experimental. (See detailed section in the paper)
Geometry. All e.s.d.'s (except the e.s.d. in the dihedral angle between two l.s. planes) are estimated using the full covariance matrix. The cell e.s.d.'s are taken into account individually in the estimation of e.s.d.'s in distances, angles and torsion angles; correlations between e.s.d.'s in cell parameters are only used when they are defined by crystal symmetry. An approximate (isotropic) treatment of cell e.s.d.'s is used for estimating e.s.d.'s involving l.s. planes.
Refinement. Refinement of *F*^2^ against ALL reflections. The weighted *R*-factor *wR* and goodness of fit *S* are based on *F*^2^, conventional *R*-factors *R* are based on *F*, with *F* set to zero for negative *F*^2^. The threshold expression of *F*^2^ > σ(*F*^2^) is used only for calculating *R*-factors(gt) *etc*. and is not relevant to the choice of reflections for refinement. *R*-factors based on *F*^2^ are statistically about twice as large as those based on *F*, and *R*- factors based on ALL data will be even larger.

### Fractional atomic coordinates and isotropic or equivalent isotropic displacement parameters (Å^2^)

**Table:**

	*x*	*y*	*z*	*U*_iso_*/*U*_eq_	
C1	−0.3844 (2)	0.29787 (18)	−0.14033 (10)	0.0352 (3)	
C2	−0.2172 (2)	0.25475 (16)	−0.09903 (9)	0.0298 (3)	
C3	−0.0448 (2)	0.14665 (16)	−0.13307 (9)	0.0292 (3)	
C4	−0.0139 (2)	0.06331 (18)	−0.21181 (10)	0.0343 (3)	
C5	0.01227 (19)	0.24707 (15)	0.00609 (9)	0.0275 (3)	
C6	0.09080 (19)	0.27024 (15)	0.07290 (9)	0.0276 (3)	
C7	0.3151 (2)	0.25405 (16)	0.18511 (9)	0.0292 (3)	
C8	0.14603 (19)	0.36850 (16)	0.21550 (8)	0.0277 (3)	
C9	0.4763 (2)	0.01342 (18)	0.30608 (10)	0.0420 (4)	
H9A	0.4424	−0.0567	0.2711	0.050*	
H9B	0.5960	−0.0498	0.3258	0.050*	
C10	0.3135 (2)	0.05787 (19)	0.38801 (10)	0.0428 (4)	
H10A	0.1927	0.1205	0.3690	0.051*	
H10B	0.2928	−0.0424	0.4210	0.051*	
C11	0.3623 (2)	0.1552 (2)	0.44647 (10)	0.0433 (4)	
C12	−0.1478 (2)	0.56270 (19)	0.33796 (10)	0.0423 (4)	
H12A	−0.1782	0.6517	0.3770	0.051*	
H12B	−0.2004	0.6090	0.2847	0.051*	
C13	−0.2516 (3)	0.4360 (2)	0.38520 (11)	0.0539 (4)	
H13A	−0.3915	0.4887	0.3985	0.065*	
H13B	−0.2281	0.3495	0.3454	0.065*	
C14	−0.1865 (3)	0.3618 (2)	0.46835 (13)	0.0552 (4)	
N1	−0.5150 (2)	0.33249 (19)	−0.17432 (10)	0.0528 (4)	
N2	0.0141 (2)	−0.00538 (19)	−0.27451 (10)	0.0527 (4)	
N3	0.4075 (2)	0.22886 (19)	0.49019 (10)	0.0591 (4)	
N4	−0.1328 (3)	0.3044 (2)	0.53217 (12)	0.0805 (6)	
S1	−0.23311 (5)	0.34738 (4)	−0.00256 (2)	0.03481 (10)	
S2	0.14881 (5)	0.11197 (4)	−0.07788 (2)	0.03252 (10)	
S3	−0.04110 (5)	0.40991 (4)	0.15451 (2)	0.03209 (10)	
S4	0.33230 (5)	0.16723 (4)	0.08462 (2)	0.03296 (10)	
S5	0.11523 (6)	0.48537 (4)	0.30581 (2)	0.03523 (10)	
S6	0.52255 (5)	0.19092 (5)	0.23438 (2)	0.03667 (10)	

### Atomic displacement parameters (Å^2^)

**Table:**

	*U*^11^	*U*^22^	*U*^33^	*U*^12^	*U*^13^	*U*^23^
C1	0.0287 (7)	0.0404 (7)	0.0378 (8)	−0.0074 (6)	−0.0108 (6)	−0.0051 (6)
C2	0.0279 (7)	0.0340 (7)	0.0304 (7)	−0.0099 (5)	−0.0106 (5)	−0.0011 (5)
C3	0.0290 (7)	0.0339 (7)	0.0276 (7)	−0.0101 (5)	−0.0094 (5)	−0.0024 (5)
C4	0.0298 (7)	0.0405 (7)	0.0339 (8)	−0.0080 (6)	−0.0107 (6)	−0.0040 (6)
C5	0.0256 (6)	0.0296 (6)	0.0279 (6)	−0.0058 (5)	−0.0082 (5)	−0.0033 (5)
C6	0.0266 (7)	0.0298 (6)	0.0265 (6)	−0.0061 (5)	−0.0071 (5)	−0.0036 (5)
C7	0.0284 (7)	0.0358 (7)	0.0247 (6)	−0.0073 (5)	−0.0090 (5)	−0.0039 (5)
C8	0.0299 (7)	0.0316 (7)	0.0232 (6)	−0.0087 (5)	−0.0079 (5)	−0.0026 (5)
C9	0.0485 (9)	0.0365 (8)	0.0406 (8)	−0.0009 (6)	−0.0209 (7)	−0.0058 (6)
C10	0.0468 (9)	0.0429 (8)	0.0410 (8)	−0.0129 (7)	−0.0177 (7)	0.0061 (7)
C11	0.0442 (9)	0.0469 (9)	0.0318 (8)	−0.0025 (7)	−0.0095 (7)	0.0002 (7)
C12	0.0417 (9)	0.0431 (8)	0.0338 (8)	0.0021 (6)	−0.0044 (6)	−0.0126 (6)
C13	0.0451 (10)	0.0769 (12)	0.0416 (9)	−0.0216 (9)	0.0003 (8)	−0.0158 (8)
C14	0.0595 (12)	0.0595 (11)	0.0473 (10)	−0.0259 (9)	0.0031 (9)	−0.0079 (9)
N1	0.0366 (8)	0.0634 (9)	0.0596 (9)	−0.0057 (6)	−0.0221 (7)	−0.0064 (7)
N2	0.0516 (9)	0.0652 (9)	0.0444 (8)	−0.0106 (7)	−0.0157 (7)	−0.0181 (7)
N3	0.0653 (11)	0.0632 (9)	0.0453 (8)	−0.0030 (8)	−0.0172 (8)	−0.0165 (7)
N4	0.1064 (17)	0.0812 (13)	0.0587 (11)	−0.0397 (12)	−0.0161 (11)	0.0099 (10)
S1	0.02631 (18)	0.0407 (2)	0.0367 (2)	−0.00195 (14)	−0.01004 (15)	−0.01111 (15)
S2	0.02509 (18)	0.03996 (19)	0.03258 (19)	−0.00337 (14)	−0.00934 (14)	−0.01026 (15)
S3	0.02685 (18)	0.03621 (19)	0.03086 (18)	−0.00055 (13)	−0.00891 (14)	−0.00827 (14)
S4	0.02534 (18)	0.0421 (2)	0.02945 (18)	−0.00093 (14)	−0.00827 (14)	−0.01089 (14)
S5	0.0400 (2)	0.03828 (19)	0.02918 (18)	−0.01030 (15)	−0.00711 (15)	−0.00961 (14)
S6	0.02798 (19)	0.0510 (2)	0.03197 (19)	−0.00714 (15)	−0.01181 (15)	−0.00518 (16)

### Geometric parameters (Å, °)

**Table:**

C1—N1	1.1341 (19)	C9—C10	1.524 (2)
C1—C2	1.4316 (19)	C9—S6	1.8232 (17)
C2—C3	1.355 (2)	C9—H9A	0.9700
C2—S1	1.7352 (15)	C9—H9B	0.9700
C3—C4	1.424 (2)	C10—C11	1.464 (2)
C3—S2	1.7417 (14)	C10—H10A	0.9700
C4—N2	1.1404 (19)	C10—H10B	0.9700
C5—C6	1.3462 (19)	C11—N3	1.136 (2)
C5—S2	1.7611 (15)	C12—C13	1.520 (2)
C5—S1	1.7648 (14)	C12—S5	1.8066 (17)
C6—S3	1.7527 (15)	C12—H12A	0.9700
C6—S4	1.7558 (14)	C12—H12B	0.9700
C7—C8	1.351 (2)	C13—C14	1.465 (3)
C7—S6	1.7553 (14)	C13—H13A	0.9700
C7—S4	1.7628 (14)	C13—H13B	0.9700
C8—S5	1.7483 (14)	C14—N4	1.136 (2)
C8—S3	1.7536 (14)		
			
N1—C1—C2	178.90 (18)	C11—C10—C9	111.21 (14)
C3—C2—C1	122.75 (13)	C11—C10—H10A	109.4
C3—C2—S1	118.32 (11)	C9—C10—H10A	109.4
C1—C2—S1	118.92 (11)	C11—C10—H10B	109.4
C2—C3—C4	123.46 (13)	C9—C10—H10B	109.4
C2—C3—S2	117.65 (11)	H10A—C10—H10B	108.0
C4—C3—S2	118.88 (10)	N3—C11—C10	177.38 (19)
N2—C4—C3	178.71 (16)	C13—C12—S5	115.32 (11)
C6—C5—S2	121.71 (11)	C13—C12—H12A	108.4
C6—C5—S1	122.52 (11)	S5—C12—H12A	108.4
S2—C5—S1	115.77 (8)	C13—C12—H12B	108.4
C5—C6—S3	122.05 (11)	S5—C12—H12B	108.4
C5—C6—S4	123.27 (11)	H12A—C12—H12B	107.5
S3—C6—S4	114.67 (8)	C14—C13—C12	112.75 (15)
C8—C7—S6	125.66 (11)	C14—C13—H13A	109.0
C8—C7—S4	117.08 (10)	C12—C13—H13A	109.0
S6—C7—S4	117.20 (8)	C14—C13—H13B	109.0
C7—C8—S5	123.02 (11)	C12—C13—H13B	109.0
C7—C8—S3	117.09 (10)	H13A—C13—H13B	107.8
S5—C8—S3	119.69 (8)	N4—C14—C13	178.8 (2)
C10—C9—S6	114.19 (10)	C2—S1—C5	94.05 (7)
C10—C9—H9A	108.7	C3—S2—C5	94.21 (7)
S6—C9—H9A	108.7	C6—S3—C8	95.71 (7)
C10—C9—H9B	108.7	C6—S4—C7	95.29 (7)
S6—C9—H9B	108.7	C8—S5—C12	102.98 (7)
H9A—C9—H9B	107.6	C7—S6—C9	100.68 (7)
			
N1—C1—C2—C3	−50 (9)	C1—C2—S1—C5	−178.05 (12)
N1—C1—C2—S1	129 (9)	C6—C5—S1—C2	−179.46 (12)
C1—C2—C3—C4	−1.2 (2)	S2—C5—S1—C2	−0.12 (8)
S1—C2—C3—C4	−179.88 (11)	C2—C3—S2—C5	0.78 (12)
C1—C2—C3—S2	177.64 (11)	C4—C3—S2—C5	179.67 (11)
S1—C2—C3—S2	−1.04 (16)	C6—C5—S2—C3	179.05 (12)
C2—C3—C4—N2	−168 (8)	S1—C5—S2—C3	−0.29 (9)
S2—C3—C4—N2	14 (8)	C5—C6—S3—C8	178.64 (12)
S2—C5—C6—S3	177.67 (7)	S4—C6—S3—C8	−2.39 (9)
S1—C5—C6—S3	−3.04 (18)	C7—C8—S3—C6	−0.40 (12)
S2—C5—C6—S4	−1.21 (18)	S5—C8—S3—C6	174.61 (8)
S1—C5—C6—S4	178.08 (7)	C5—C6—S4—C7	−177.40 (12)
S6—C7—C8—S5	5.36 (19)	S3—C6—S4—C7	3.65 (9)
S4—C7—C8—S5	−171.73 (7)	C8—C7—S4—C6	−4.05 (12)
S6—C7—C8—S3	−179.81 (8)	S6—C7—S4—C6	178.61 (8)
S4—C7—C8—S3	3.10 (16)	C7—C8—S5—C12	−162.25 (12)
S6—C9—C10—C11	−62.05 (14)	S3—C8—S5—C12	23.05 (10)
C9—C10—C11—N3	−17 (4)	C13—C12—S5—C8	73.13 (13)
S5—C12—C13—C14	59.88 (18)	C8—C7—S6—C9	93.08 (14)
C12—C13—C14—N4	−35 (11)	S4—C7—S6—C9	−89.84 (9)
C3—C2—S1—C5	0.68 (12)	C10—C9—S6—C7	−72.76 (12)
